# Interspecific Nest Parasitism by *Pseudabispa paragioides*, a Solitary Australian Wasp

**DOI:** 10.1673/031.010.14120

**Published:** 2010-09-22

**Authors:** Robert W. Matthews, Janice R. Matthews

**Affiliations:** Department of Entomology, University of Georgia, Athens, GA 30602 USA

**Keywords:** exploitation, interspecific competition, kleptoparasitism, lethal fighting, prey theft, provisioning, social parasitism, usurpation

## Abstract

In morphology, coloration, and size, *Pseudabispa* wasps (Hymenoptera: Vespidae: Eumeninae) closely resemble mason wasps in the genus *Abispa,* and their distributions overlap. Although these two genera are among the largest solitary wasps in Australia, the biology of *Pseudabispa* was not previously known. Field observations from near Katherine, Northern Territory, strongly suggest that *P. paragioides* (Meade-Waldo) females attack and kill female *A. ephippium* (Fabricius) and usurp their nests, then appropriate cells, mass provision them with caterpillars acquired by theft from still other nests, and close them with mud taken from the host nest. Despite an abundance of potentially available cells in nests of three other large solitary wasps common at the same site, *P. paragioides* was found associated only with nests of *A. ephippium.* This unusual report of apparently forcible and lethal interspecific nest takeover for a non-social wasp parallels behaviors previously known only from socially parasitic eusocial Hymenoptera. Exploitation by *P. paragioides* may help explain why its host displays some of the most highly developed parental care known in any solitary eumenid, and why its nests are spaced widely from one another.

## Introduction

Parasitic exploitation is a common phenomenon among animals and occurs in many contexts. It is thought to be a significant force promoting speciation, hence biodiversity (Summers et al. 2003). Among insects most parasites exploit the physiology of the host, but there are many other cases in which a parasite commandeers host-created resources for its own reproduction.

The nests of bees and wasps are valuable resources that may be exploited by a variety of parasites and inquilines that do not construct nests. Kleptoparasitism, or food theft, describes the situation where thieving individuals steal or intercept food intended for their host, benefiting themselves directly (Iyengar 2008). Brood parasitism (also termed “stealth parasitism” describes cases in which the parasite surreptitiously enters the host nest and deposits an egg, thereby exploiting both the shelter and food of the host (O'Neill 2001).

Among solitary bees and wasps nest usurpation is widespread, but it appears to occur primarily between conspecifics (Field 1992). Interspecific nest takeovers are uncommon (McIntosh 1996; Gess and Gess 1988). In many instances takeovers occur when the owner is away, but aggressive intraspecific usurpations also occur as in the sphecid wasp, *Sphex ichneumoneus* (L.) (Brockmann et al. 1979). Lethal interspecific fighting for nest ownership is far more characteristic of the eusocial Hymenoptera, where it is expressed as various forms of social parasitism (Wilson 1971).

Van der Vecht (1960) described the eumenid wasp genus *Pseudabispa,* recognizing four species from Australia, and commented that its resemblance to another eumenid genus, *Abispa,* “is so striking, that even experienced entomologists have overlooked the morphological differences between the corresponding species of the two genera. Very probably it will be difficult to distinguish these wasps in the field, and although some authors have published observations on the life history of certain species of *Abispa,* the bionomics of *Pseudabispa* appear to be completely unknown.”

Initially the authors of this paper were also confused by the superficial resemblance of the two genera whose adults were both relatively common at the study site, but whose nests were difficult to locate. The confusion grew when *Pseudabispa paragioides* (Meade-Waldo) were reared from otherwise *Abispa ephippium* (Fabricius) nests. Here the first biological information for *Pseudabispa,* based on field observations of *P. paragioides,* is provided.

## Methods and Materials

*Pseudabispa paragioides* was studied at Charles Darwin University's Northern Territory Rural College campus, 15K N. of Katherine (14° 22.545′ S, 132° 09.403′ E), where it was found to exploit nests of *Abispa ephippium* located on various campus buildings always in rain-sheltered, low-light niches. The drive-through carport and ground floor laundry area of a “Queenslander” style residence (see Figure 2 in Matthews and Matthews 2009) comprised the principal study site.

Study dates were 27 November to 10 December 2004 and 17 November to 4 December 2007. In both years, to allow individual recognition, wasps of both sexes of both species flying through the area or visiting a small pond nearby were opportunistically captured and marked with Liquid Paper® on the thorax and/or abdomen using unique combinations of spots of white, green, and silver.

In 2007, a focal nest constructed by *Abispa ephippium* that had been chosen for detailed behavioral observations in another study (Matthews and Matthews 2009) serendipitously proved also to be a focus of *P. paragioides.* This nest was situated in a dark corner of a bookcase below the lowermost shelf 6 cm above the floor and was observed for a total of 58.25 hours over 15 days, of which the last six days were occupied by a marked female of *P. paragioides.* A clean piece of paper was placed on the floor below the nest to catch any dropped materials and cleared of any such debris at the end of each day.

**Figure 1.**
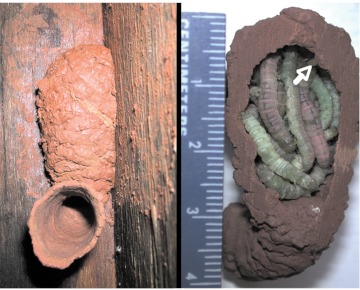
(Left) A single-celled nest of *Abispa ephippium* was occupied by a *Pseudabispa paragioides* female when discovered in 2004. (Right) After removal from substrate, the nest was found to contain an egg (arrow) and 20 caterpillars mass provisioned by the *P. paragioides* female. High quality figures are available online.

Voucher material was initially identified by comparison with authoritatively identified specimens deposited in the CSIRO Insect Collection in Canberra, ACT, and subsequently verified by specialists (see acknowledgements). Specimens from this study are deposited in the CSIRO Insect Collection, the American Museum of Natural History, and the University of Georgia (Fattig Entomology Museum).

## Results

In 2004 we captured, marked, and released 47 wasps; two of these were *P. paragioides.* Both were males; the first was caught on 30 November and the second on 2 December. Both were flying in the carport, one at 09:12 hours and the other at 15:45 hours. The latter was recaptured twice, once at a nearby small pond six days later and again a day later in the carport.

On 7 December a female *P. paragioides* on a nest was discovered, the only one found in 2004. The wasp was actively provisioning a well-hidden, single-celled *A. ephippium* nest that, upon dissection, was found to contain 20 paralyzed caterpillars and an egg (Figure 1). The 3 mm long egg was suspended on a 0.5 mm thread 2mm from the back wall of the cell. Based on the small number of adults seen, the nearly complete absence of any nesting activity and the discovery of a teneral adult male in a cell in each of two *A. ephippium* nests dissected on 30 November, we speculated that *P. paragioides* had just started to emerge and become active toward the end of the study in 2004.

In 2007 the nesting season appeared to begin somewhat earlier than in 2004. An *A. ephippium* nest dissected on 21 November 2007 contained two cells with *P. paragioides* late pupae. Fewer *A. ephippium* wasps and active nests were found in 2007 than in 2004 (Matthews and Matthews 2009); 30 wasps were captured, marked, and released. Of these, 15 *were P. paragioides*: 14 females and a single male. Finding this number was unexpected, considering how uncommon they had been in 2004. All but two of the *P. paragioides* were captured in the morning; *P. paragioides* were seldom seen in the afternoon (1200 – 1700 hours). On one morning (1 December) five *P. paragioides* females searching in the carport area were marked.

Detailed observations of a single marked *P. paragioides* female wasp and the active focal nest of *A. ephippium* she usurped in 2007 are the basis for the remainder of the results. On 25 November, a *P. paragioides* female that had been captured in the carport and marked on 18 November was observed twice approaching the focal *A. ephippium* nest in such a direct manner as to suggest prior knowledge of it. Although the *P. paragiodes* female was not observed to ever alight at the nest, four days later she was found dead on the carport floor not far from the focal nest.

On 26 November, a *P. paragioides* female caught flying in the carport area near the focal *A. ephippium* nest was marked and released. The next day, this marked female was recorded flying/searching in the immediate focal nest vicinity or landing close to the nest three different times. Twice she interacted briefly with the marked resident *A. ephippium* female who lunged toward the intruder, causing it to quickly fly away. Otherwise, the *A. ephippium* female spent most of that day applying mud to the nest exterior.

**Figure 2.**
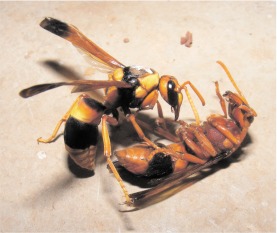
A marked female of *Pseudabispa paragioides* (top) in a battle with the female of *Abispa ephippium* (below) that owned the focal nest observed in 2007; shortly after this photograph was taken, the *A. ephippium* female died and *P. paragioides* occupied her nest. Note the similarity in size and coloration of the two species. High quality figures are available online.

On 27 November, at 07:45 the marked *A. ephippium* female observed over the previous eight days was discovered lying lethargically on the floor about 60 cm from its nest and the female *P. paragioides* marked two days earlier was on the nest. The apparently recovering *A. ephippium* female was placed back onto the nest and a series of fierce fights followed that were documented with video and still photos (Figure 2) in which the two wasps grappled and fell to the floor where they appeared to be repeatedly trying to sting one another. At the sudden conclusion of each battle the *A. ephippium* female appeared completely paralyzed, but partially revived 30–50 seconds later. She never recovered enough to fly, but could walk and cling to the nest when placed on it. However, by noon that day the *A. ephippium* female was dead on the floor, and the marked *P. paragioides* female was resident on nest.

**Figure 3.**
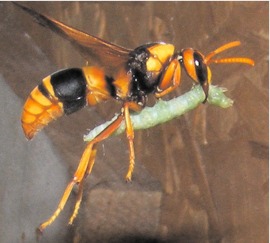
*Pseudabispa paragioides* returning to the focal nest with caterpillar prey. Note that her front legs are not used to support the prey during transport. High quality figures are available online.

**Figure 4.**
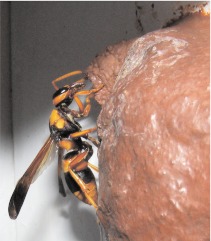
*Pseudabispa paragioides* obtaining mud from the remains of the nest funnel originally constructed by *Abispa ephippium.* The mud was used to close a newly provisioned cell elsewhere on the nest (out of view). High quality figures are available online.

By 08:00 the next morning, the *P. paragioides* female was bringing prey, carrying them as shown in Figure 3. Subsequent nest removal and dissection revealed that the cell being provisioned was the original *A. ephippium* cell 5. By late morning the *P. paragioides* female had brought several caterpillars and then switched to capping the cell. For this she used mud taken from the rim of the old nest funnel, biting off small pieces (Figure 4), moistening them with regurgitated water, and then applying the mud to crudely close the cell opening. Periodically she left the nest for brief periods, apparently to gather water and then returned and resumed mud borrowing until the cell was fully closed. The wasp was never observed to bring mud pellets to the nest.

After closing this cell, the wasp began to work on the original but now empty cell 6, vigorously cleaning out debris that accumulated on the floor below the nest. This occurred over two extended bouts, during which she repeatedly (40+ times) entered head first and then backed out far enough to drop debris over the cell entrance. Following this, she worked at the entrance rim, rapidly opening and closing her jaws as part of repeated head-bobbing movements while she slowly rotated her body around the entrance rim. She was not obviously applying anything and her jaw tips appeared to barely contact the nest, but she spent a lot of time repeating this behavior, which was punctuated by breaks to crawl into the cell and remove more small pieces of debris and by brief grooming episodes. She gripped the nest primarily with her hind tarsi. This cell preparation stage extended over two hours.

**Figure 5.**
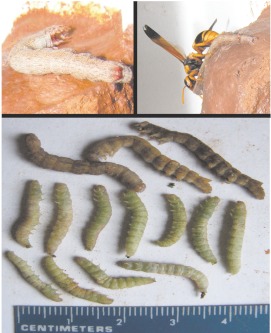
(Above) When paralyzed caterpillars were placed successively on top of the focal nest, *Pseudabispa paragioides* immediately moved them into the cell with her other prey. (Below) Upon dissection, the mass provisioned cell contained 14 prey, including the three experimentally offered caterpillars (at top). High quality figures are available online.

By 07:30 the next morning (two days since takeover) the *P. paragioides* female was again bringing prey and stuffing them into a cell. One caterpillar had been dropped to the floor below the nest and abandoned before we arrived. On the following day the wasp was still working at the nest in a visually inaccessible area, but three holes (probably representing emergence of prior cell inhabitants) could be felt with a finger. On the floor below the nest lay a new pile of mud chips and old cell debris.

On the fourth day (07:20) the marked *P. paragioides* female was still in residence on the nest. She brought in at least seven caterpillars storing them in the original cell 3. At one point while she was in cell 3 head first, another marked *P. paragioides* female came directly to the nest, landed, and went straight to cell 3. When she encountered the abdomen of the resident female she abruptly flew away. About 30 minutes later when the resident was away the intruder female again appeared, landed on the nest, and crawled straight to cell 3 and quickly removed two caterpillars (one was probably stuck to the other) and attempted to fly off with them. While escaping, she collided with a research camera, fell to the floor, and dropped the caterpillars. When the resident female returned a few minutes later (with nothing), her behavior suggested that she recognized that there had been an intruder. She entered the cell and then rapidly exited, repeating this behavior at least 15 times.

Because prey theft seemed to be the major source of the caterpillars used by *P. paragioides,* a simple experiment was performed. The now-resident *P. paragioides* female readily accepted paralyzed larvae of *Achaea argilla* (Noctuidae: Catocalinae) taken from a newly provisioned cell of a nest of *Delta latreillei,* even though these caterpillars were considerably larger than the prey she had previously stocked. When they were individually draped over the top of the nest (Figure 5, top), the female quickly discovered them and took three caterpillars in succession, stuffing them into the cell atop her other prey. All were later recovered from this cell, which contained a total of 14 caterpillars (Figure 5, bottom).

Once when the marked female was returning with a caterpillar, an unidentified miltogrammine fly was observed following her. It landed on the wall adjacent to the nest while the female stored her caterpillar and departed. The fly then immediately flew to the nest and entered the cell being provisioned, then exited quickly and flew away making an audible high pitched whine. Upon nest dissection, two small maggots were found attached to one of the caterpillars in the provisioned cell.

The *P. paragioides* female was clearly most active in the morning; the afternoon pattern on all days of observation revealed little activity at the nest, with the female often being absent for extended periods. Nonetheless, provisioning was rapid at a rate of about one cell mass-provisioned per day.

The single cell comprising the 2004 nest held 20 caterpillars; three cells of the 2007 focal nest contained 8, 11, and 14 prey, respectively, while the fourth held a nearly mature *P. paragioides* larva. Prey were only lightly paralyzed and continued to defecate after being stored in the cells (one of the caterpillars in Figure 5 is voiding a fecal pellet). As Figures 1, 3, and 5 illustrate, *P. paragioides* appeared to specialize on relatively few species of small caterpillars in the superfamily Pyraloidea. However, due to the aforementioned propensity for prey theft, apparent prey specificity probably more accurately reflected the prey preferences of those species from whom they stole. Australian caterpillars are notoriously difficult to identify. However, adult moths that were subsequently reared from lightly paralyzed prey that pupated were identified as “likely to be” Crambidae, “probably” a species of Pyraustinae and Pyralidae, or “probably“ a species of Phycitinae; two caterpillars from another cell pupated, but desiccated before they could emerge.

During observations of the 2007 focal nest two other nests were discovered nearby that were being actively provisioned by other marked *P. paragioides* females. Each was observed bringing several small caterpillars, again primarily during the morning, but unfortunately both of these nests were inaccessible. One nest had been built in the interior space of one of the cement blocks that formed the house foundation; the wasp was gaining access via an old drill hole. The other nest was behind the framing above a door, probably in the space between the header boards; this wasp was entering through a small gap above the doorframe.

Though both male mate location tactics and copulation were frequently observed for *A. ephippium* during both years of the study (Matthews and Matthews 2009), sexual behavior by *P. paragioides* was never observed. The fact that so few males were seen or captured in the carport area in 2007 where at least three females were actively provisioning different nests suggests that mating may occur at sites other than the nesting area.

## Discussion

In morphology, coloration, and size, mason wasps in the genera *Abispa* and *Pseudabispa* are superficially very similar to one another, and mimetic-appearing species pairs occur together in parts of Australia (van der Vecht 1960). Although both genera are commonly collected, the biology of *Abispa* was only recently studied in detail (Matthews and Matthews 2004, 2009). While larger sample sizes would be desirable, the fact that the observations reported here constitute the first information relating to the biology of the genus *Pseudabispa* justifies placing these findings on record, even though they are limited and the interpretations speculative.

In addition to direct observations of two *Abispa* nests usurped by *P. paragioides,* circumstantial evidence was found for the regular association of these two species in the form of three instances in which dissected *A. ephippium* nests contained cells with immature *P. paragioides.* Perhaps, like so many other species of solitary wasps, *P. paragioides* simply reuses cells from which the original occupant has emerged. However, observations described here strongly suggest that these wasps forcibly displace the original nest owner and remain associated with their usurped nest, reusing and mass provisioning empty or incomplete cells. Whether females also break into sealed *Abispa* cells and replace the contents with their own will require additional study.

Numerous dissections of both older and recently completed nests of three other species of large mason and mud dauber wasps common at the study site (*Delta latreillei* [Hymenoptera: Eumenidae], *Sceliphron formosum,* and *S. laetum* [Hymenoptera: Sphecidae]) disclosed no cells that contained developing *P. paragioides,* though several other secondary nest inhabitants were found. Although these other solitary wasp nests offered abundant opportunity for *Pseudabispa* to reuse cells, none were so occupied, a fact that is further suggestive of a tight association between *Abispa* and *Pseudabispa.*

If confirmed, the regularly fatal nature of the aggression between *P. paragioides* and *A. ephippium* for possession of the latter's nest would be unusual between two species of nonsocial hymenopterans. In many respects the observed behavior of the female *P. paragioides* was similar to that seen in socially parasitic vespine wasps. For example, queens of *Vespula squamosa* search out young nests of *V. maculifrons* and aggressively attack and kill the *V. maculifrons* queen, then usurp the nest; this social wasp differs only in that it also makes use of the emerging host workers' labor to expand the nest for its own progeny during a transition period following usurpation (MacDonald and Matthews 1975). *Polistes atrimandibularis* also engages in violent takeovers of nests of its main host species, *P. biglumis* (Schwammberger 2001). Several lineages of socially parasitic ants also are known, but host colony usurpations among ants usually involve mimicking and manipulating host semiochemicals rather than employing brute force (Lenoir et al. 2001).

By definition solitary species cannot exhibit social parasitism, but there is no reason to suppose that parasites in non-social taxa would not have taken similar evolutionary pathways. Among those who study socially parasitic eusocial Hymenoptera, there has been much discussion concerning whether they follow Emery's Rule, which posits that social parasites and their hosts are closely related (Lowe et al. 2002; Huang and Dornhaus 2008). In many ants Emery's Rule does seem to apply, but in other eusocial Hymenoptera, such as vespine wasps, studies have shown that social parasites are not closely related (Carpenter and Perera 2006). As noted above, *Pseudabispa* and its host *Abispa* appear superficially quite similar in size and coloration. Moreover, the two genera overlap substantially in their distribution in Australia and New Guinea. However, no phylogenetic analysis has been done for these genera, and there is no *a priori* basis for regarding *Pseudabispa* as closely related to *Abispa* (J.M. Carpenter, American Museum of Natural History, Division of Invertebrate Zoology, New York, NY, personal communication).

Other examples of fatal fighting over nest ownership between different nonsocial insect species occur in thrips (Thysanoptera) and gall wasps (Cynipidae), both of which alter plant growth to form domiciles. The dwellings produced by thrips upon Australian acacias are often contested by other species of nondomicile producing thrips (Bono 2007 and references therein); some of these species simply take over abandoned domiciles, but at least one species fights and sometimes kills the domicile owner. Similar examples are known among gall wasps; some inquiline species kill their gall-inducing host (Ronquist 1994). Many forms of intra- and inter-specific nest exploitation are also known to occur in various lineages of solitary bees and wasps (Michener 1974; O'Neill 2001), but lethal fights between different species for possession of a nest are not documented.

The selective pressure exerted by usurping *Pseudabispa* also may have driven certain aspects of *Abispa* biology and evolution, particularly since the costs of lethal exploitation are considerably higher than those resulting from the various forms of kleptoparasitism. Additionally, the fact that both very young and relatively mature nests of *A. ephippium* were usurped suggests that the selective pressure exerted by *P. paragioides* is not limited to a particular host nest stage or time frame. As noted by Matthews and Matthews (2009), the nests of *A. ephippium* are fortress-like, isolated, and well hidden. They never occur in aggregations like many mud dauber wasp species (e.g., *Sceliphron formosum,* which nested abundantly at the study site). Moreover, *A. ephippium* invest in unusually high levels of maternal care, monitoring their developing larvae frequently while progressively provisioning prior to the final cell closures. When exposed to other potential parasitic insects, *Abispa* responded with intensified inspection behaviors. They also displayed swift and violent responses during chance encounters with intruders. The discovery of a recently deceased *P. paragioides* female near the focal *Abispa* nest further suggests that *P. paragioides* do not necessarily always win such contests and that *A. ephippium* likely can successfully deter attempted *P. paragioides* usurpations. Whether either host or parasite has special morphological adaptations, such as thickened cuticles, awaits further study.

The discovery that *P. paragioides* females opportunistically steal prey from other nests, including those of conspecific females as well as *A. ephippium* (Matthews and Matthews 2009) is similar to observations of other solitary wasps (see review in O'Neill 2001). If *P. paragioides* is ultimately found to have lost the ability to capture and paralyze prey and relies entirely on opportunistic theft, then it could also be termed a true brood parasite (*sensu* Field 1992) because its larval sustenance would depend upon caterpillars originally captured by other wasp species, even though these provisions are first relocated to a new cell. If confirmed by further research, it will be another way in which *Pseudabispa* is unusual, because obligatory prey theft is unknown in any other solitary wasps. Interestingly, *Polistes atrimandibularis* are also reported to provision their offspring both with brood stolen from other nests of their host and with prey taken from host wasps returning to these nests (Schwammberger 2001).

Although venom injection could not be confirmed during the fights witnessed, the sudden end of the battles and subsequent partial recovery strongly suggest that *Abispa* was stung during their grappling. While simple exhaustion could account for this behavior, the *Abispa's* response was quite similar to that seen in battles between resident and potentially usurping queens of socially parasitic *Vespula* wasps (Matthews and Matthews 1979). Further knowledge of the biochemical composition of *Pseudabispa's* venom and its effect on other vespid wasps would be of interest in this regard.

Taken together, these observations on *Pseudabispa* suggest a unique kleptoparasitic lifestyle that at the very least is facultative, and may be obligatory. Whether *P. paragioides* females ever independently construct a nest or forage for mud or prey will require further study, but it seems unlikely. Moreover, circumstantial evidence suggests that females of *P. paragioides* probably also challenge conspecifics for ownership of the relatively rare *Abispa* nests and that successive replacements are possible on any given nest depending on the outcomes of such contests.
